# Expression of concern: Promising antimicrobial and antibiofilm activities of reduced graphene oxide-metal oxide (RGO-NiO, RGO-AgO, and RGO-ZnO) nanocomposites

**DOI:** 10.1039/d4ra90062f

**Published:** 2024-05-28

**Authors:** Sherif Elbasuney, Gharieb S. El-Sayyad, Hesham Tantawy, Amr H. Hashem

**Affiliations:** a Head of Nanotechnology Research Center, Military Technical College (MTC), Egyptian Armed Forces, Kobry Elkobbah Cairo 262-111 Egypt; b Chemical Engineering Department, Military Technical College (MTC), Egyptian Armed Forces, Kobry Elkobbah Cairo 262-111 Egypt; c Drug Radiation Research Department, National Center for Radiation Research and Technology (NCRRT), Egyptian Atomic Energy Authority (EAEA) Nasr City Cairo 11787 Egypt Gharieb.Elsayaad@eaea.org.eg; d Botany and Microbiology Department, Faculty of Science, Al-Azhar University Cairo 11884 Egypt amr.hosny86@azhar.edu.eg

## Abstract

Expression of concern for ‘Promising antimicrobial and antibiofilm activities of reduced graphene oxide-metal oxide (RGO-NiO, RGO-AgO, and RGO-ZnO) nanocomposites’ by Sherif Elbasuney *et al.*, *RSC Adv.*, 2021, **11**, 25961–25975, https://doi.org/10.1039/D1RA04542C.

*RSC Advances* is publishing this expression of concern in order to alert readers that concerns have been raised regarding the reliability of the antimicrobial activity photos in Fig. 10 and SEM images in Fig. 13. An investigation is underway, and an expression of concern will continue to be associated with the article until a final outcome is reached.

Laura Fisher

22nd May 2024

Executive Editor, *RSC Advances*

## Supplementary Material

